# Widespread tissue distribution of transcriptionally active, clonally expanded HIV-1 proviruses despite suppressive antiretroviral therapy

**DOI:** 10.1172/JCI190824

**Published:** 2025-04-22

**Authors:** Hiromi Imamichi, Ven Natarajan, Francesca Scrimieri, Mindy Smith, Yunden Badralmaa, Marjorie Bosche, Jack Hensien, Thomas Buerkert, Weizhong Chang, Brad Sherman, Kanal Singh, H. Clifford Lane

**Affiliations:** 1 Clinical and Molecular Retrovirology Section, Laboratory of Immunoregulation and Infectious Diseases, National Institute of Allergy and Infectious Diseases, NIH, Bethesda, Maryland, USA.; 2 Frederick National Laboratory for Cancer Research, Frederick, Maryland, USA.

**Keywords:** AIDS/HIV, Infectious disease, Virology, Molecular biology, Molecular pathology

## Abstract

Evidence of HIV-1 associated DNA and RNA can be found in multiple tissues despite adequate anti-retroviral therapy.

**To the Editor:** Despite the success of antiretroviral therapy (ART) in suppressing plasma viremia levels to below 40 copies/mL, HIV-1 persists in tissue reservoirs, presenting a major barrier to efforts toward a cure. However, the extent to which residual viral expression differs between individuals with controlled versus active viral replication remains unclear.

HIV-DNA and cell-associated (CA) HIV-RNA were detected across various autopsy tissues from persons with HIV (PWH) (Figure 1A). CA HIV-RNA was detected in 12 out of 13 donors and in 27 of the 30 different tissues examined. There were no statistically significant differences between the ‘Active’ group (HIV-RNA detected in peripheral blood at the time of autopsy, *n* = 5, participants 1–5) and the ‘Suppressed’ group (HIV-RNA not detected in peripheral blood at the time of autopsy, *n* = 8, participants 6–13) (Fisher’s exact test, *P* > 0.10 for all comparisons, Supplemental Table 1; supplemental material available online with this article; https://doi.org/10.1172/JCI190824DS1). Additionally, no universal hot spots were identified for HIV-DNA and/or CA HIV-RNA.

HIV-1 proviruses with the potential to encode replication-competent viruses were detected in 9 out of 10 donors for whom HIV-1 sequence data were available (Supplemental Figure 1A). This was seen in 4.3%–7.1% of sequences in the ‘Active’ group and 0%–17.4% of sequence in the ‘Suppressed’ group. A breakdown of the number of full-length intact HIV-DNA is shown in Supplemental Figure 1, B–D, demonstrating their random distribution across different tissue sites, with no observed differences between the ‘Active’ and ‘Suppressed’ groups.

Clonally expanded HIV-1 provirus sequences were found in all 10 donors with available HIV-1 provirus sequence data (Figure 1B). On average, 47.4% (120 of 253) of sequences in the ‘Active’ group (*n* = 3); and 56.0% (923 of 1,649) of sequences in the ‘Suppressed’ group (*n* = 7) were found in expanded clones. No significant difference was seen between the 2 groups (*P* > 0.99). Of note, the number of distinct clonally expanded HIV-1 provirus sequences identified was proportional to the total number of HIV-1 provirus sequences obtained (*r* = 0.97, *P* < 0.01; Figure 1C). Given that the number of sequences obtained for studying clonal expansions of cells harboring HIV-1 proviruses is not standardized, caution is advised when making conclusions about the contributions of clonal expansions to the overall pool of HIV-1 proviruses. Our findings indicate that a large number of cells harboring HIV-DNA originated from clonal expansions.

Expanded clones harboring HIV-1 proviruses are generated through the proliferation of HIV-1–infected CD4^+^ T cells in response to antigenic or homeostatic forces in cells that survived their initial infection. Identical HIV-1 provirus sequence types found in different tissue sites reflect the distribution or trafficking of expanded CD4^+^ T cell clones and could be observed in all donors. As one representative example, 71 distinct clones harboring an HIV-1 provirus were identified in participant 12 in the ‘Suppressed’ group. Twelve of these clones were distributed across 20 tissue compartments, including peripheral blood (Figure 1D). Clonally expanded proviral genomes were detected in 19 of the 20 tissue sites, except for the gastroesophageal (GE) junction, where only 2 amplicons were generated (Figure 1E). Of note, 65% of the proviral sequences were associated with clonal expansions, all of which were classified as defective and not able to encode replication-competent viruses. However, 47% contained an intact open reading frame. There was evidence of transcription in bone marrow and intestinal tissues, including the duodenum, ileum, ascending colon, and transverse colon (Figure 1D and Supplemental Figure 2, A and B). Additional analyses of the remaining nine donors demonstrate that clonal populations are shared across tissues (Supplemental Figure 3, Supplemental Table 1).

The persistence of HIV-1 proviruses throughout the body has been demonstrated (1–3). In the present study, outside of peripheral blood, similar levels of HIV-DNA and HIV-RNA expression were detected in autopsy samples from PWH with either ‘Active’ or ‘Suppressed’ HIV-1 replication. These data imply that a substantial seeding of tissues with cells harboring transcriptionally active proviral DNA can be seen in the setting of HIV-1 infection despite ART. Limitations of the study include: (a) the lack of sampling from less-expected tissues such as adipose, skin, bone, and skeletal muscle, (b) the absence of longitudinal viral load data prior to autopsy, (c) the limited number of participants, and (d) lack of information on immune cell composition across tissue types. This study provides the first description of transcriptionally active defective HIV-1 proviruses in various tissue compartments; however, their contribution to systemic immune dysfunction remains an area of active investigation. While the majority of these HIV-RNAs likely originate from cells harboring defective proviruses, these findings, combined with the fact that some transcriptionally active defective proviruses express viral proteins (4), highlight one of the challenges in achieving an HIV-1 cure and provide the intriguing possibility that HIV-1 infection could lead to sustained alterations in the human genome.

## Supplementary Material

Supplemental data

Supporting data values

## Figures and Tables

**Figure 1:**
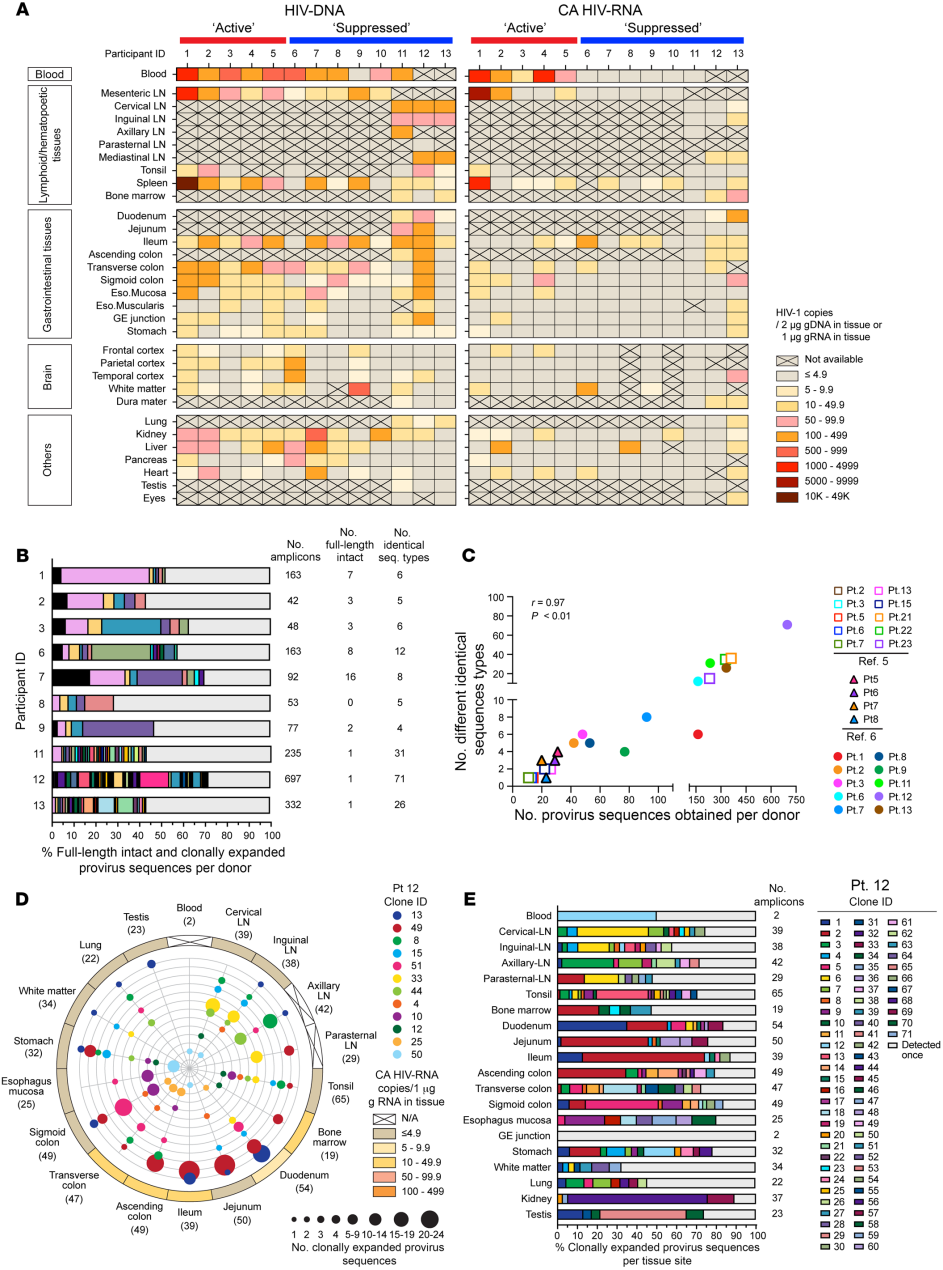
HIV-DNA and cell-associated HIV-RNA were measured in all 13 donors across 31 tissue compartments and peripheral blood, with most HIV-1 proviruses representing clonally expanded sequences. (**A**) Levels of HIV-DNA (copies/2 μg of genomic DNA) and cell-associated (CA) HIV-RNA (copies/1 μg of total RNA) in the ‘Active’ group (HIV-1 levels ≥ 4.9 copies/2 μg gDNA or 1 μg gRNA) in blood at autopsy, *n* = 5) and ‘Suppressed’ group (HIV-1 levels < 4.9 copies/2 μg gDNA or 1 μg gRNA), *n* = 8). HIV-DNA ranged from 5 to 11,340 copies; CA HIV-RNA ranged from 7 to 7,814 copies. (**B**) Proportions of full-length intact (black) and clonally expanded (colored) HIV-1 proviruses in each donor. (**C**) Correlation between the number of distinct identical HIV-1 sequence types and the total sequences per donor (Spearman test) (5, 6). (**D**) In participant 12, 12 distinct HIV-1 clones were distributed across ≥ 4 tissue compartments. Numbers in parentheses indicate total sequences per tissue. The outer ring reflects CA HIV-RNA levels using the same gradient as **A**. Similar data for participants 1–3, 6–9, 11, and 13 are in Supplemental Figure 3. (**E**) Proportions of clonally expanded defective HIV-1 proviruses in 19 tissue compartments and blood from participant 12.

## Version 1 04/29/2025

In-Press Preview

## Version 2 06/16/2025

Electronic publication
