# Supplementary material for: Widespread tissue distribution of transcriptionally active, clonally expanded HIV-1 proviruses despite suppressive antiretroviral therapy
Source: J Clin Invest. 2025 Apr 22;135(12):e190824. doi: 10.1172/JCI190824 (PMC12165783; doi:10.1172/JCI190824)
Supplement: Supplemental data [file jci-135-190824-s248.pdf]

Supplementary Materials and Methods

Details of study participants and tissue specimens.

Of 13 participants, ten donors were identified through the National Disease Research Interchange (NDRI); three donors had been enrolled in National Institutes of Health Institutional Review Board-approved HIV-1 clinical research protocols at the time of death. Informed consent was obtained from a family member or legal representative. Characteristics of the study participants are shown in Table S1. Autopsies for participants 1-10 were conducted within 24 hours of death. For participants 11-13, autopsies were delayed by 24 hours or more due to consent acquisition or travel arrangement delays. Tissues were snap-frozen in liquid nitrogen immediately after collection and stored at -80°C until processing. During autopsy, tissue samples were obtained from distinct anatomical sites, with biopsy sizes typically ranging from 3 to 5 mm^3. To minimize sample error within an individual tissue, samples were pooled from different locations.

Quantification and amplification of HIV-DNA and cell-associated (CA) HIV-RNA

Total HIV-DNA and cell-associated (CA) HIV-RNA were isolated from autopsy tissue samples using MasterPure Complete DNA and RNA Purification Kit (Biosearch Technologies), Gentra Puregene Cell Kit (Qiagen) and the RNeasy Mini kit (Qiagen) per manufacturer's instructions, respectively. Levels of HIV-DNA and CA HIV-RNA copy numbers were quantified with 7900HT Fast-Real time PCR system (ThermoFisher Scientific) using primers and probes as previously reported (7, 8). HIV-1 copy numbers are expressed as copies per milliliter of blood. The limit of detection of the quantitative PCR (qPCR) is 5 copies/2 µg of genomic DNA for HIV-DNA and 5 copies/1 µg of total RNA for CA HIV-RNA. Genomic DNA and cDNA were subjected to limiting dilution before amplification with a near full-length PCR as previously described (5).

Sequence analyses

Single-molecule direct sequencing was performed using the 3500xL Genetic Analyzer (Applied Biosystems by ThermoFisher Scientific) with a BigDye Terminator v3.1 Cycle Sequencing Kit. Intactness of the HIV-1 genome was assessed with the HIVAlign program (www.hiv.lanl.gov)(9). AliView (v1.26)(10) and was used to generate sequence alignments. “Full-length intact” HIV-1s are defined as species that are intact in length and possess all nine intact protein-coding genes (gag, pol, env, nef, tat, rev, vpr, vif, vpu). “Full-length defective” HIV-1s are intact in length but contain out-of-frame indels, premature stop codons, hypermutations, or inversions. “Defective” HIV-1s are less than intact in length and may contain large internal deletions with/without out-of-frame indels, premature stop codons, hypermutations, or inversions.

Statistical Analyses

Nonparametric rank-based correlations (Spearman) and Fisher’s exact test were performed for analyses using the Prism software (version 9.3.1). Figures were constructed using the Prism and Adobe Illustrator software.

Data availability

The sequences reported in this paper have been deposited in the GenBank database under accession numbers PV115194-PV116225.

Acknowledgements

The authors thank Drs. Frank Maldarelli and Stephen Hewitt of the National Cancer Institute for providing tissue samples from donors 11-13. This work was funded in part through the Division of Intramural Research of the National Institute of Allergy and Infectious Diseases, NIH, and in part with federal funds from the National Cancer Institute, NIH, under Contract No.

HHSN261200800001E. The content of this publication does not necessarily reflect the views or policies of the Department of Health and Human Services, nor does mention of trade names, commercial products, or organizations imply endorsement by the US Government. The views expressed in this article are those of the authors and do not necessarily represent the views of the National Institute of Allergy and Infectious Diseases (NIAID) or Frederick National Laboratory for Cancer Research. We acknowledge the use of tissues procured by the National Disease Research Interchange (NDRI) with support from NIH grant U42OD11158.

References cited in the Methods section

7. Palmer S, et al. New real-time reverse transcriptase-initiated PCR assay with single-copy sensitivity for human immunodeficiency virus type 1 RNA in plasma. J Clin Microbiol. 2003;41:4531-6.
8. Somsouk M, et al. The immunologic effects of mesalamine in treated HIV-infected individuals with incomplete CD4R T cell recovery: a randomized crossover trial. PLoS One. 2014;9:e116306.
9. Gaschen B, et al. Retrieval and on-the-fly alignment of sequence fragments from the HIV database. Bioinformatics. 2001;17:415-8.
10. Larsson A. AliView: a fast and lightweight alignment viewer and editor for large datasets. Bioinformatics. 2014;30:3276-8.

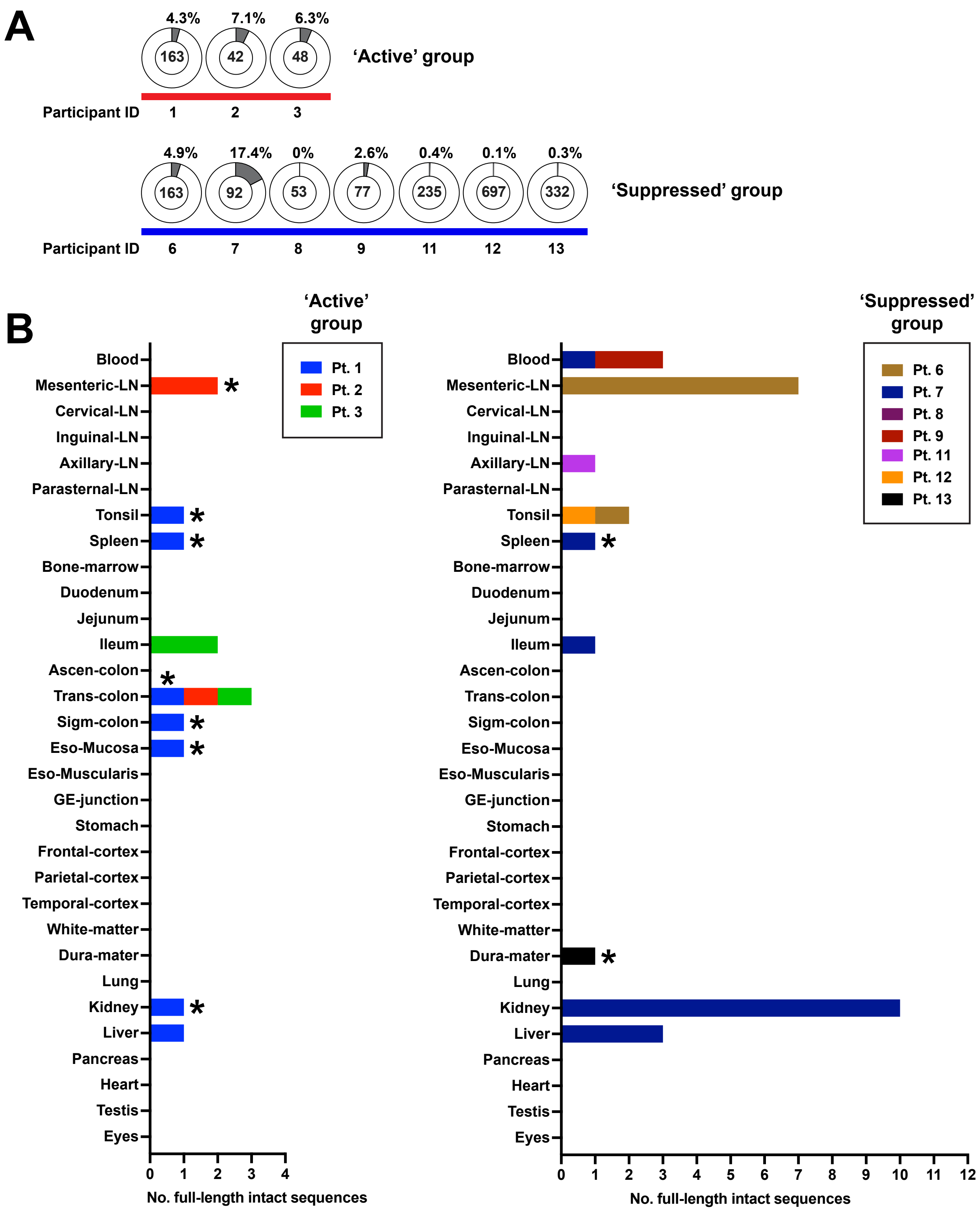
A
4.3%
7.1%
6.3%
163
42
48
'Active' group
Participant ID
1
2
3
4.9%
17.4%
0%
2.6%
0.4%
0.1%
0.3%
163
92
53
77
235
697
332
'Suppressed' group
Participant ID
6
7
8
9
11
12
13
B
'Active' group
Pt. 1
Pt. 2
Pt. 3
'Suppressed' group
Pt. 6
Pt. 7
Pt. 8
Pt. 9
Pt. 11
Pt. 12
Pt. 13
Blood
Mesenteric-LN
Cervical-LN
Inguinal-LN
Axillary-LN
Parasternal-LN
Tonsil
Spleen
Bone-marrow
Duodenum
Jejunum
Ileum
Ascen-colon
Trans-colon
Sigm-colon
Eso-Mucosa
Eso-Muscularis
GE-junction
Stomach
Frontal-cortex
Parietal-cortex
Temporal-cortex
White-matter
Dura-mater
Lung
Kidney
Liver
Pancreas
Heart
Testis
Eyes
No. full-length intact sequences

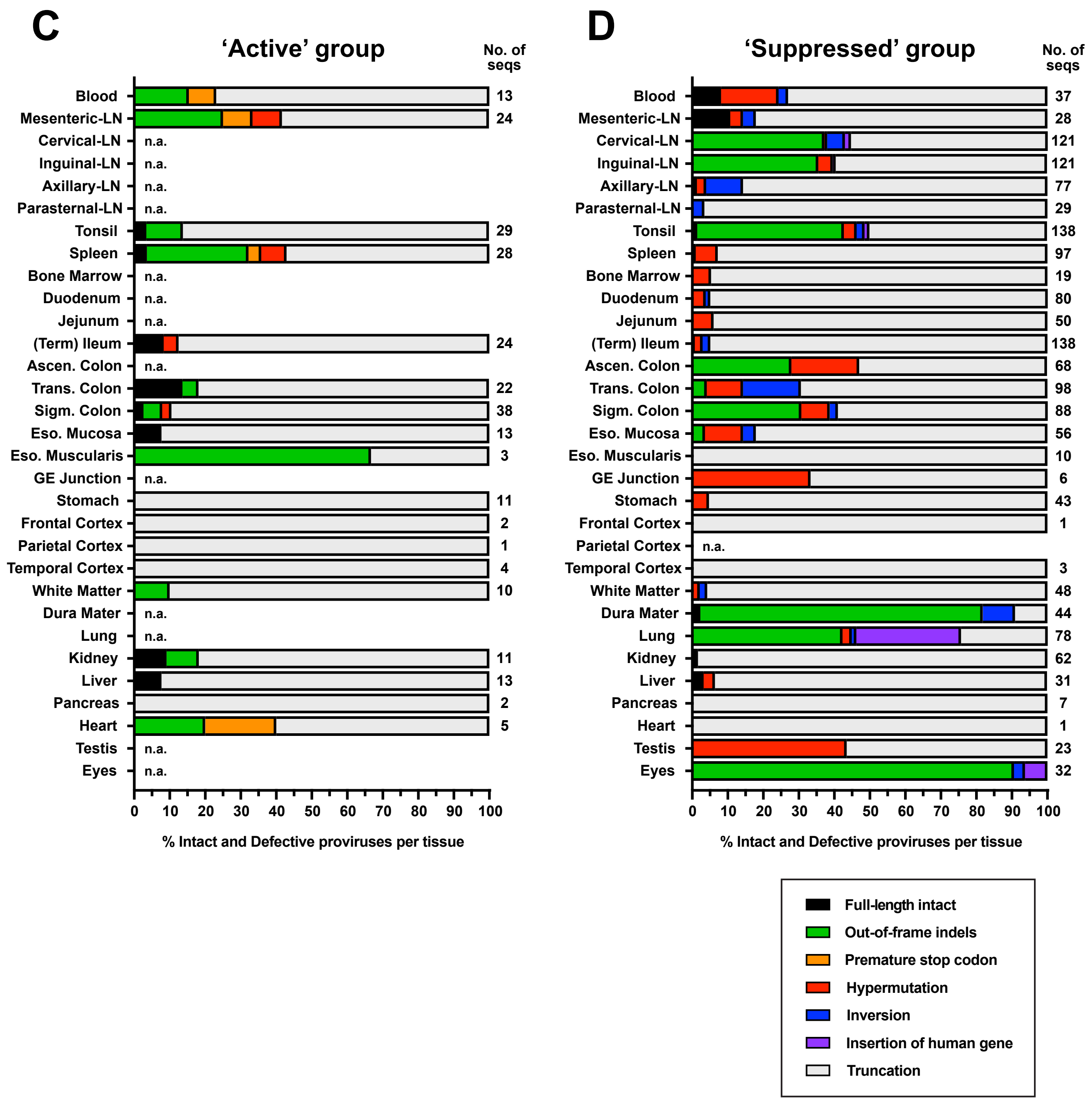

Figure S1. Tissue specific enrichment of full-length intact HIV-1 proviruses was not observed. (A) On average, 5.9% (13/253) of proviruses in the 'Active' and 3.7% (29/1649) of proviruses in the 'Suppressed' groups were full-length intact, capable of producing replication-competent viruses. No difference (p=0.18) was seen between the two groups. Mann-Whitney non-parametric test. **(B)** The number of full-length intact HIV-1 provirus

sequences were plotted for each tissue compartment. The data were analyzed separately for two groups: the 'Active' group, consisting of PWH donors with detectable HIV-RNA in peripheral blood at the time of autopsy (n=5), and the 'Suppressed' group, consisting of PWH donors with undetectable HIV-RNA in peripheral blood at the time of autopsy (n=8). Full-length intact HIV-1 proviruses associated with more than five HIV-RNA copies per microgram of genomic RNA in tissue are marked with asterisks. Of the 16 intact proviruses identified in participant 7 in 'Suppressed' group, 15 were found to belong to expanded clones. (C) Percentages of full-length intact and defective HIV-1 proviruses for each tissue compartment of the 'Active' group, consisting of PWH donors with detectable HIV-RNA in peripheral blood at autopsy (n=5); and (D) the 'Suppressed' group, consisting of PWH donors with undetectable HIV-RNA in peripheral blood at autopsy (n=8). The total number of sequences obtained for each tissue compartment is shown under 'No. of seqs'. The color codes used in the plots are explained in the key.

A

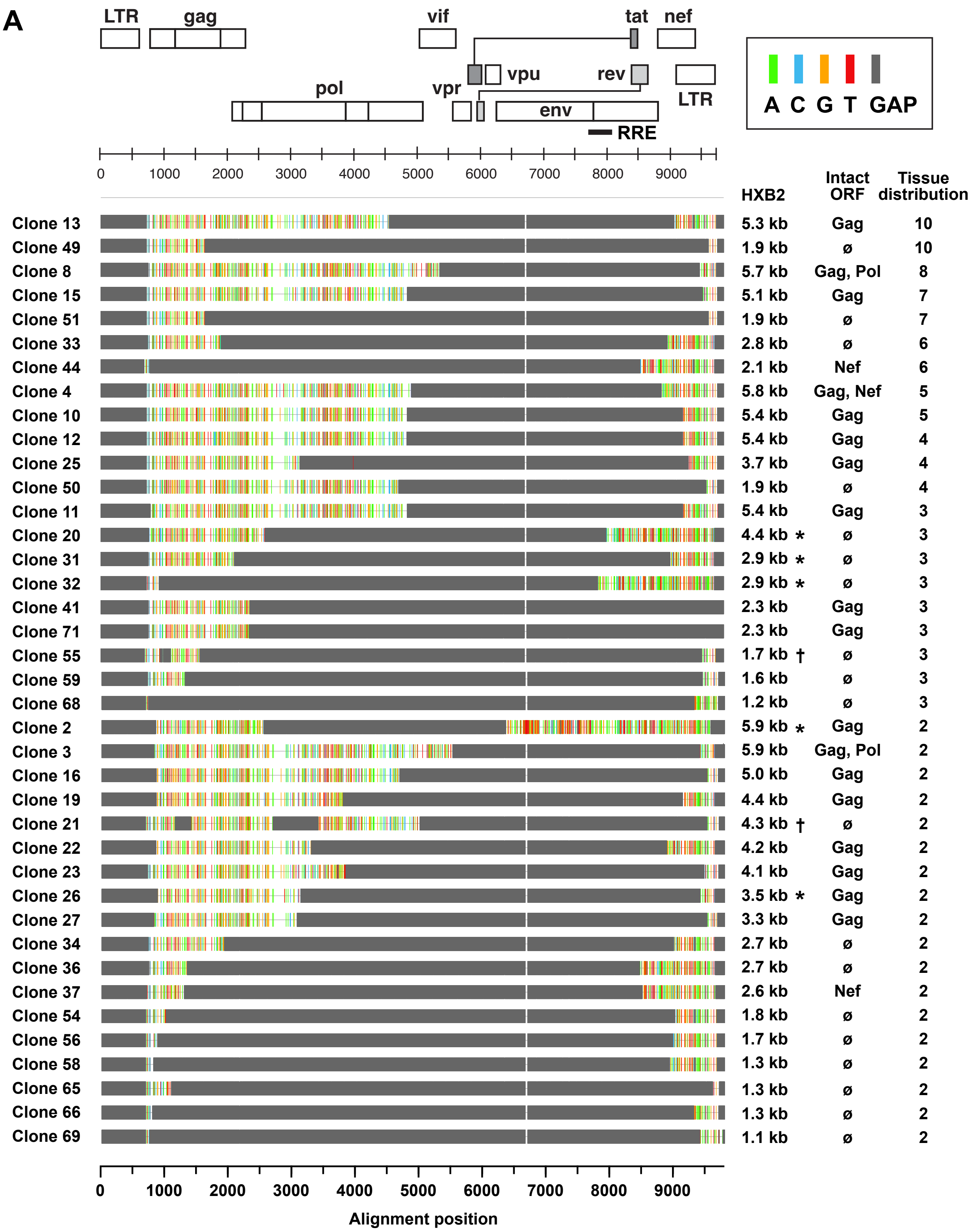

LTR
gag
vif
tat
nef
vpu
rev
pol
vpr
LTR
env
RRE
A C G T GAP
0 1000 2000 3000 4000 5000 6000 7000 8000 9000
HXB2
Intact ORF
Tissue distribution
Clone 13 5.3 kb Gag 10
Clone 49 1.9 kb ø 10
Clone 8 5.7 kb Gag, Pol 8
Clone 15 5.1 kb Gag 7
Clone 51 1.9 kb ø 7
Clone 33 2.8 kb ø 6
Clone 44 2.1 kb Nef 6
Clone 4 5.8 kb Gag, Nef 5
Clone 10 5.4 kb Gag 5
Clone 12 5.4 kb Gag 4
Clone 25 3.7 kb Gag 4
Clone 50 1.9 kb ø 4
Clone 11 5.4 kb Gag 3
Clone 20 4.4 kb * ø 3
Clone 31 2.9 kb * ø 3
Clone 32 2.9 kb * ø 3
Clone 41 2.3 kb Gag 3
Clone 71 2.3 kb Gag 3
Clone 55 1.7 kb † ø 3
Clone 59 1.6 kb ø 3
Clone 68 1.2 kb ø 3
Clone 2 5.9 kb * Gag 2
Clone 3 5.9 kb Gag, Pol 2
Clone 16 5.0 kb Gag 2
Clone 19 4.4 kb Gag 2
Clone 21 4.3 kb † ø 2
Clone 22 4.2 kb Gag 2
Clone 23 4.1 kb Gag 2
Clone 26 3.5 kb * Gag 2
Clone 27 3.3 kb Gag 2
Clone 34 2.7 kb ø 2
Clone 36 2.7 kb ø 2
Clone 37 2.6 kb Nef 2
Clone 54 1.8 kb ø 2
Clone 56 1.7 kb ø 2
Clone 58 1.3 kb ø 2
Clone 65 1.3 kb ø 2
Clone 66 1.3 kb ø 2
Clone 69 1.1 kb ø 2
0 1000 2000 3000 4000 5000 6000 7000 8000 9000
Alignment position

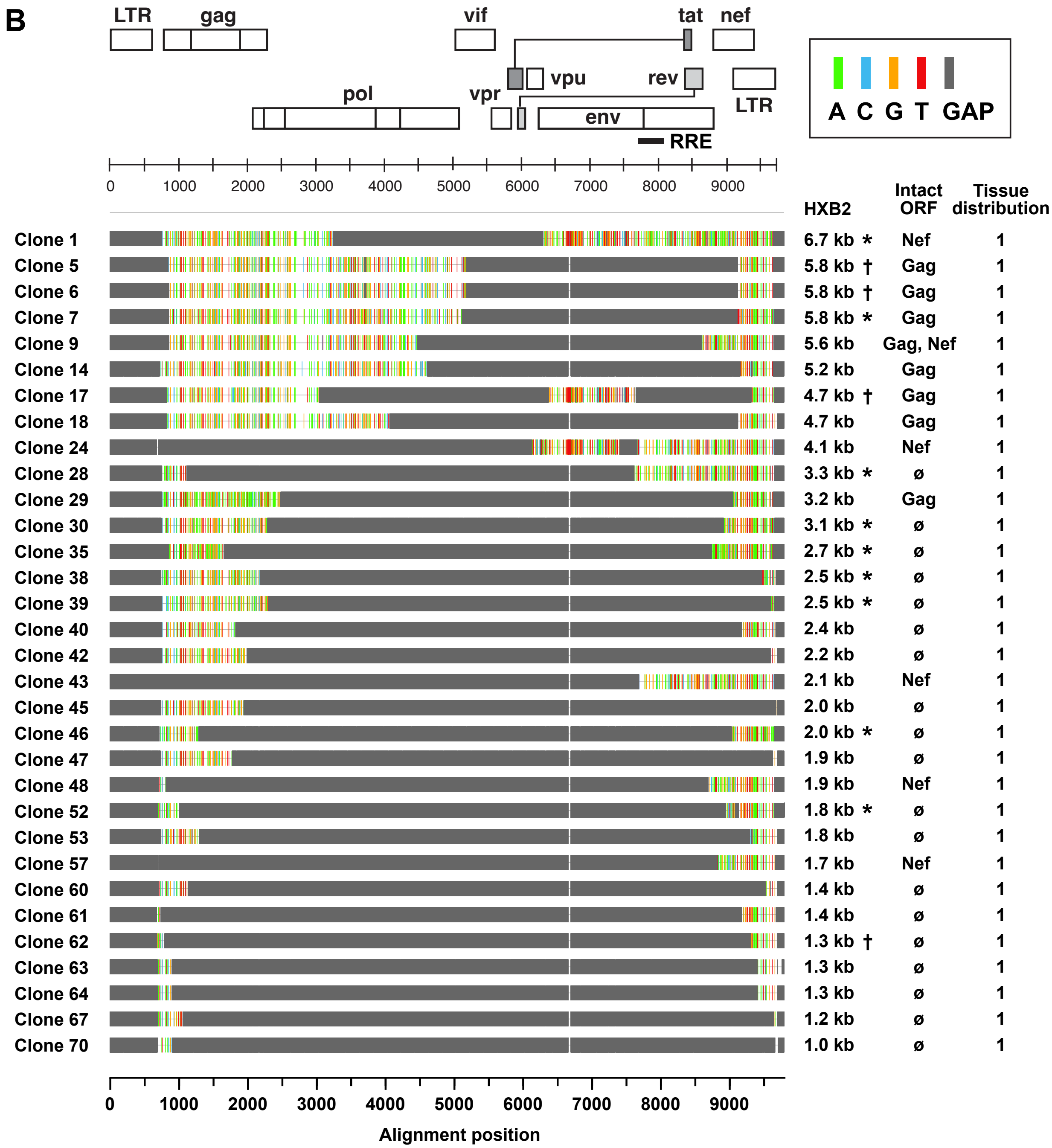

Figure S2. All expanded HIV-1 provirus clone sequences identified in the tissues of participant 12 were defective due to large internal deletions but retained intact Gag and Nef ORFs. The Highlighter analysis shows nucleotide substitutions compared to the HIV-1 HXB2 reference. A schematic diagram of the HIV-1 genome of the HXB2 strain is

displayed at the top. The estimated size (in kilobases), presence of intact open reading frames (ORFs), and tissue distribution for each expanded clone are indicated on the right. *Hypermutant. †Contained inversion.

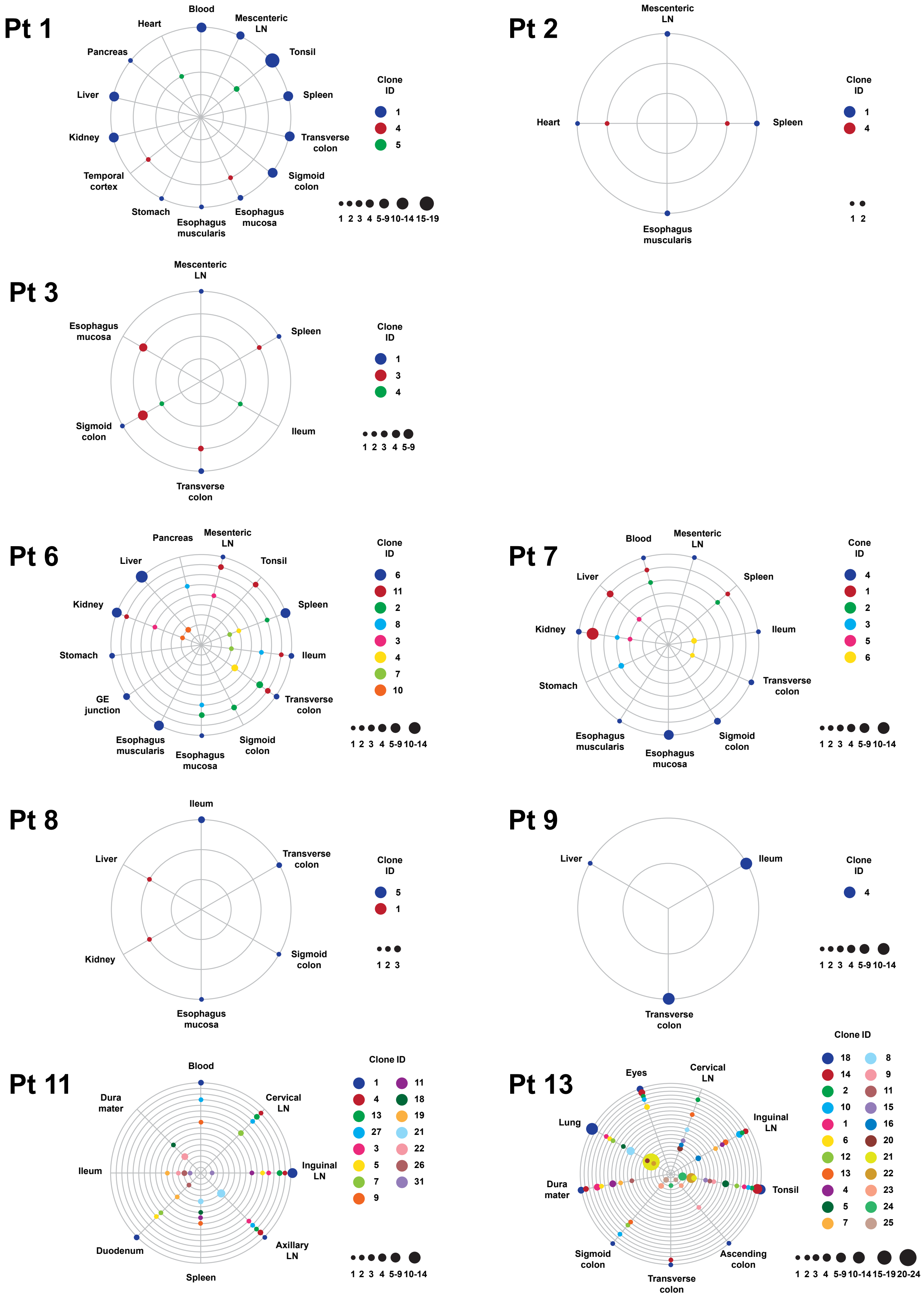
Pt 1
Blood
Mescenteric LN
Tonsil
Spleen
Transverse colon
Sigmoid colon
Esophagus mucosa
Esophagus muscularis
Stomach
Temporal cortex
Kidney
Liver
Pancreas
Heart
Clone ID
1
4
5
1 2 3 4 5-9 10-14 15-19
Pt 2
Mescenteric LN
Heart
Spleen
Esophagus muscularis
Clone ID
1
4
1 2
Pt 3
Mescenteric LN
Spleen
Ileum
Transverse colon
Sigmoid colon
Esophagus mucosa
Clone ID
1
3
4
1 2 3 4 5-9
Pt 6
Pancreas
Mesenteric LN
Tonsil
Liver
Kidney
Spleen
Stomach
Ileum
GE junction
Transverse colon
Esophagus muscularis
Esophagus mucosa
Sigmoid colon
Clone ID
6
11
2
8
3
4
7
10
1 2 3 4 5-9 10-14
Pt 7
Blood
Mesenteric LN
Liver
Spleen
Kidney
Ileum
Stomach
Transverse colon
Esophagus muscularis
Esophagus mucosa
Sigmoid colon
Cone ID
4
1
2
3
5
6
1 2 3 4 5-9 10-14
Pt 8
Ileum
Liver
Transverse colon
Kidney
Sigmoid colon
Esophagus mucosa
Clone ID
5
1
1 2 3
Pt 9
Liver
Ileum
Transverse colon
Clone ID
4
1 2 3 4 5-9 10-14
Pt 11
Blood
Dura mater
Cervical LN
Ileum
Inguinal LN
Duodenum
Axillary LN
Spleen
Clone ID
1
4
13
27
3
5
7
9
11
18
19
21
22
26
31
1 2 3 4 5-9 10-14
Pt 13
Eyes
Cervical LN
Lung
Inguinal LN
Dura mater
Tonsil
Sigmoid colon
Ascending colon
Transverse colon
Clone ID
18
14
2
10
1
6
12
13
4
5
7
8
9
11
15
16
20
21
22
23
24
25
1 2 3 4 5-9 10-14 15-19 20-24

Figure S3. Clonal relationships across different tissue compartments in the body. The distribution of distinct expanded HIV-1 provirus clones across more than four different tissue compartments is shown for 'Active' group: PWH donors with HIV-RNA detected in peripheral blood at the time of autopsy (Pts 1-3) and 'Suppressed' group: HIV-RNA not detected in peripheral blood at the time of autopsy (Pts 6-9, 11 and 13).

Table S1. Study participants

Participant ID	Age	Gender	Cause of death	Years since HIV-1 diagnosis[1]	CD4 count (cells/µL) prior to death	Total HIV-RNA in peripheral blood (copies/1 µg gRNA) at time of death	HIV-RNA in plasma (copies/mL) prior to death	Group
1	45	M	Cardiac arrest	n.a.	n.a.	3550	n.a.	Active
2	58	M	n.a.	n.a.	n.a.	198	n.a.	Active
3	61	M	n.a.	n.a.	n.a.	15	n.a.	Active
4	57	M	Cardiac arrest	n.a.	n.a.	1341	n.a.	Active
5	52	M	Cardiac arrest	n.a.	n.a.	59	n.a.	Active
6	59	M	Cardiac arrest	n.a.	n.a.	<4.9	n.a.	Suppressed
7	58	M	Sepsis	n.a.	n.a.	<4.9	n.a.	Suppressed
8	55	M	Hepatic encephalopathy	n.a.	n.a.	<4.9	n.a.	Suppressed
9	52	M	Cardiac arrest	n.a.	n.a.	<4.9	n.a.	Suppressed
10	54	M	Multiorgan failure	n.a.	n.a.	<4.9	n.a.	Suppressed
11	67	M	Diffuse pulmonary fat embolism	37	575	<4.9	<20	Suppressed
12	27	M	Diffuse alveolar damage	6	7	<4.9	<40	Suppressed
13	52	M	Pulmonary edema	8	480	<4.9	<40	Suppressed

[1] Estimated based on medical records.

The low CD4 count of 7 in Pt 12 was likely due to chemotherapy.

n.a., not available
